# Corneal power evaluation after myopic corneal refractive surgery using artificial neural networks

**DOI:** 10.1186/s12938-016-0243-5

**Published:** 2016-11-15

**Authors:** Robert Koprowski, Michele Lanza, Carlo Irregolare

**Affiliations:** 1 Department of Biomedical Computer Systems, Faculty of Computer Science and Materials Science, Institute of Computer Science, University of Silesia, ul. Będzińska 39, 41-200 Sosnowiec, Poland; 2 Dipartimento Multidisciplinare di Scienze Mediche, Chirurgiche e Odontoiatriche, Seconda Università di Napoli, Naples, Italy; 3 Centro Grandi Apparecchiature, Seconda Università di Napoli, Naples, Italy

**Keywords:** Signal processing, Neural networks, Corneal power, Refractive surgery, IOL power calculation

## Abstract

**Background:**

Efficacy and high availability of surgery techniques for refractive defect correction increase the number of patients who undergo to this type of surgery. Regardless of that, with increasing age, more and more patients must undergo cataract surgery. Accurate evaluation of corneal power is an extremely important element affecting the precision of intraocular lens (IOL) power calculation and errors in this procedure could affect quality of life of patients and satisfaction with the service provided. The available device able to measure corneal power have been tested to be not reliable after myopic refractive surgery.

**Methods:**

Artificial neural networks with error backpropagation and one hidden layer were proposed for corneal power prediction. The article analysed the features acquired from the Pentacam HR tomograph, which was necessary to measure the corneal power. Additionally, several billion iterations of artificial neural networks were conducted for several hundred simulations of different network configurations and different features derived from the Pentacam HR. The analysis was performed on a PC with Intel^®^ Xeon^®^ X5680 3.33 GHz CPU in Matlab^®^ Version 7.11.0.584 (R2010b) with Signal Processing Toolbox Version 7.1 (R2010b), Neural Network Toolbox 7.0 (R2010b) and Statistics Toolbox (R2010b).

**Results and conclusions:**

A total corneal power prediction error was obtained for 172 patients (113 patients forming the training set and 59 patients in the test set) with an average age of 32 ± 9.4 years, including 67% of men. The error was at an average level of 0.16 ± 0.14 diopters and its maximum value did not exceed 0.75 dioptres. The Pentacam parameters (measurement results) providing the above result are tangential anterial/posterior. The corneal net power and equivalent k-reading power. The analysis time for a single patient (a single eye) did not exceed 0.1 s, whereas the time of network training was about 3 s for 1000 iterations (the number of neurons in the hidden layer was 400).

## Background

After the introduction of the excimer laser in the 1983, refractive surgery had a huge development and the number of patients who undergo this surgery is still growing [1]. Many of these patients had developed a significant cataract, therefore underwent phacoemulsification and intraocular lens (IOL) implantation [2]. The most important factor affecting accuracy of IOL calculating in these cases is the corneal power evaluation [3]. One of most diffuse devices for keratometry (corneal refraction, measurement of the radius of the anterior corneal surface curvature) is Pentacam HR. The Oculus Pentacam HR™ is a rotating Scheimpflug video camera that generates images from the anterior surface of the cornea to the posterior surface of the lens [4–6]. The number of images acquired from the Scheimpflug camera is closely dependent on the type of the Pentacam version. It can range from a few to a dozen of images, e.g. it can be performed at angles of 48°–228°, 55°–235°, 62°–242°, etc. [7, 8]. In addition to this, the following information is also obtained: corneal thickness, tangential curvature, axial/sagittal curvature, elevation, true next power, keratomic power deviation, anterior chamber depth, refractive power, refractive pachymetry, equiv K-reading power, total corneal refractive power [9, 10]. These information are typically obtained for patients before refractive surgery. Then, the tests are repeated after surgery. If the patient further develop a cataract, there is the needing of corneal power measurement to perform IOL power calculation. In naïve eyes, this type of measurement able to provide no, or very small, residual refractive defect after lens implantation so there is no need to wear eyeglasses [11–13]. However, the situation is quite different in the case of cataract patients who have been previously subjected to myopic refractive eye surgery: in these patients, the Oculus Pentacam and the other available devices do not allow for fully correct IOL power calculation [14, 15]. Therefore, many authors [16–18] are looking for an algebraic method using formulas (e.g. interpolation and approximation) [19, 20], which would help to reduce the residual defect. However, even if these methods improved a lot the accuracy of IOL power calculation, there still are improvement needing [9]. The relationship between the performed measurements (corneal thickness, tangential curvature, axial/sagittal curvature, elevation, true next power, keratomic power deviation etc.) and the lens power after corneal refractive surgery [21–24] is not precisely defined [25–36], so this article proposes the use of artificial intelligence methods. Of the various known methods of artificial intelligence, the authors have proposed artificial neural networks. In addition to other methods such as discriminant analysis, decision trees, k-menas [37, 38] and naive Bayes classifier [39–42], neural networks enable corneal power prediction on the basis of measurements performed with the Pentacam device. The proposed proprietary analysis algorithm and the results are presented later in this article.

## Materials

**Fig. 1:**
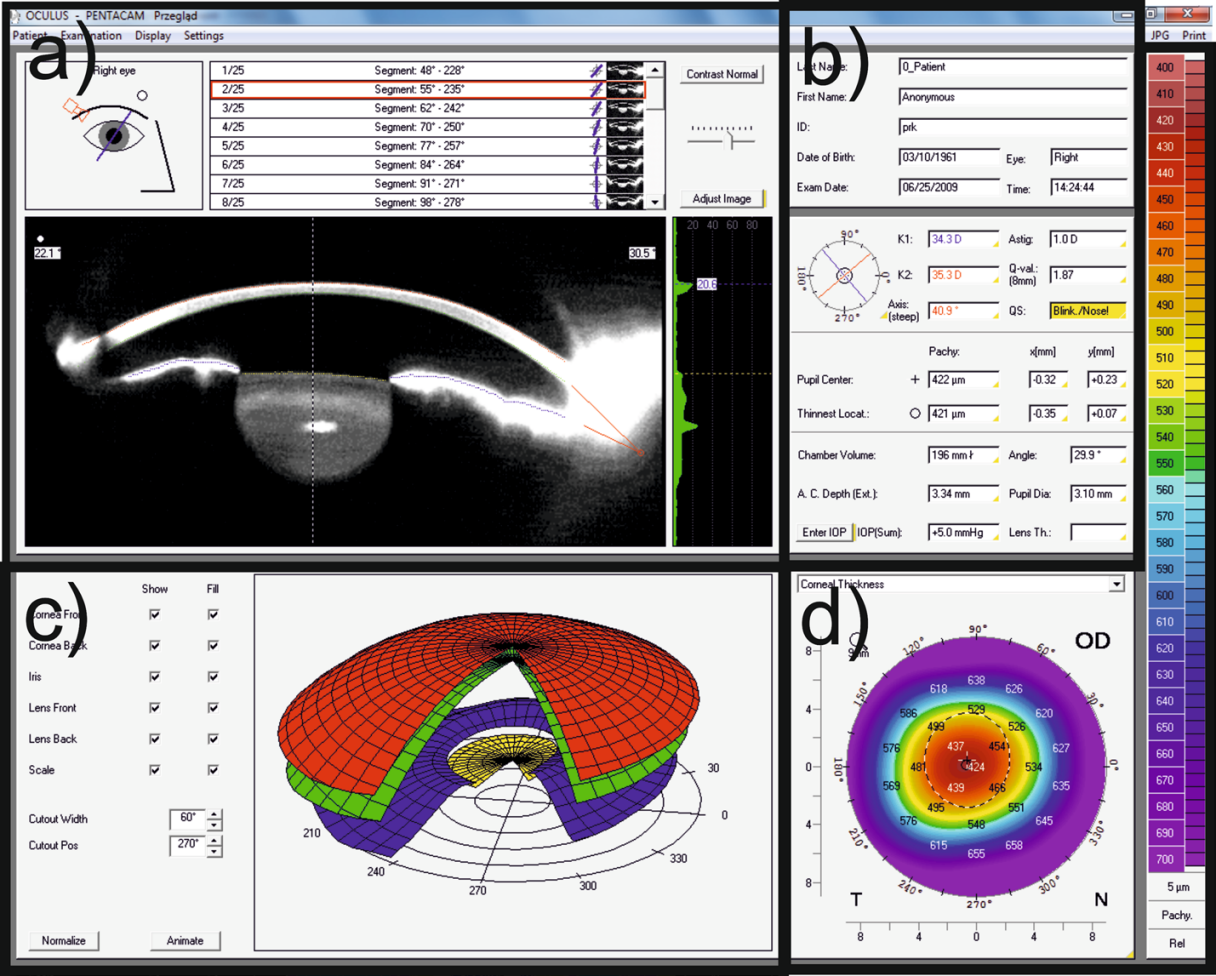
Sample result of measuring the corneal power in the Pentacam device: **a** the results of measurement using the rotating Scheimpflug video camera, **b** cornea front and back, lens front and back, **c** anonymised patient data, **d** corneal thickness

Input data were obtained from 172 patients aged between 19 and 55 years (mean age of 32 years with the standard deviation of 9.4 years), including 67% of men. The study was conducted within routine tests performed in patients treated surgically in Centro Grandi Apparecchiature, Seconda Università di Napoli, Napels, Italy. All the patients were informed about the study, which was conducted in accordance with the Declaration of Helsinki. The measurements were made using the Pentacam HR keratometer with 1–19r11 software version (Fig. 1 shows examples of the results obtained) before refractive surgery, 1 month after the surgery and 3 months later. The following characteristics were measured: gender—feature *w*(1), age—feature *w*(2), left/right eye—feature *w*(3), cornea front/back-features *w*(4) and *w*(5) as the mean values of measurements for the angle of 90° and 180°, tangential anterior/posterior-features *w*(6) and *w*(7), analogously the mean values for the measurement in 4 points (similarly for the other features), net power of the cornea—feature *w*(8), total corneal refractive power—*w*(9), equiv K-reading power—feature *w*(10). They were acquired three times, namely prior to refractive surgery, 1 month after the surgery and 3 months later. Sample results obtained before the surgery for 5 patients are shown in Table 1. The mean, minimum and maximum values as well as standard deviation of the mean of the measured parameters for all patients are shown in Table 2. In addition to these data, the optical power of the cornea was calculated after the adjustment and correction of Pentacam errors—feature *w*(11) (Tables 1, 2). Feature *w*(11) will further constitute ground truth for the proposed structure of neural networks. The proposed new algorithm was implemented on a PC with Intel^®^ Xeon^®^ X5680 3.33 GHz CPU in Matlab^®^ Version 7.11.0.584 (R2010b) with Signal Processing Toolbox Version 7.1 (R2010b), Neural Network Toolbox 7.0 (R2010b) and Statistics Toolbox (R2010b).

**Table 1:** The values of measured parameters for 5 patients (all units, unless otherwise stated, are in dioptres)

Feature	Patient 1	Patient 2	Patient 3	Patient 4	Patient 5
Male/female—*w*(1)	F	M	F	M	M
Age—*w*(2)	48	46	28	30	28
Eye left/right—*w*(3)	L	R	R	L	R
Cornea front					
90°	45.7	41.5	42.7	45.1	46.1
180°	48.4	41.5	43.1	46.8	47.3
*w*(4)—mean	47	41.5	42.9	45.9	46.7
Cornea back					
90°	−6.4	−5.8	−5.9	−6.3	−6.5
180°	−7	−6.2	−6.1	−6.9	−7.1
*w*(5)—mean	−6.7	−6	−6	−6.6	−6.8
Tangential anterior					
45°	46.8	41	43.2	45.1	45.9
90°	46.4	41	43.3	45.2	45.7
135°	48.3	41.9	43.3	47.2	46.9
180°	46.5	43.1	44.1	45.9	45.1
*w*(6)—mean	47	41.75	43.47	45.85	45.9
Tangential posterior					
45°	−7.4	−6.2	−6.2	−6.6	−7
90°	−7.3	−6.2	−6.1	−6.9	−7.1
135°	−7.4	−6.3	−6.2	−7.3	−7.4
180°	−6.9	−6.5	−6.4	−6.7	−6.8
*w*(7)—mean	−7.25	−6.3	−6.22	−6.87	−3.5
Net power of the cornea					
45°	44.7	40.3	41.7	44	45
90°	44.3	40.5	42.6	43.8	44.4
135°	47	39.7	41.2	45.2	46
180°	46.6	40.6	42.2	45.4	45.7
*w*(8)—mean	45.65	40.28	41.93	44.6	45.28
Total corneal refractive power					
45°	46	40.7	42.9	45.3	46.6
90°	45.7	41.9	43.5	45.3	46.4
135°	49.2	41.1	42.5	47.1	48.2
180°	48.6	41.8	43.4	47.2	47.7
*w*(9)	47	41	43	46	47
Equiv K-reading power					
90°	45.8	41.7	43.2	45.2	46
180°	48	42.1	43.8	46.4	46.7
*w*(10)—mean	46.9	41.9	43.5	45.8	46.4
Corrected corneal power (ground truth) *w*(11)	38	37	37.65	41.275	39.7

**Table 2:** The mean, minimum and maximum values as well as standard deviations of the mean of the measured parameters for all 172 patients (the values of the feature *w*(1) are expressed in years, the values of the other features are expressed in dioptres)

Value	Feature								
	*w*(2)	*w*(4)	*w*(5)	*w*(6)	*w*(7)	*w*(8)	*w*(9)	*w*(10)	*w*(11)
Minimum	19	39.8	−8	1.7	−19.2	38.5	40	40.4	28.6
Maximum	55	50	6.3	37.3	3.57	49.5	52	47.4	50.45
Mean ± std	32 ± 9.4	43.4 ± 1.4	−6.1 ± 1.4	43.4 ± 2	−6.4 ± 1.29	42.3 ± 1.5	44 ± 1.6	43.7 ± 1.3	40 ± 3.9

## Methods

An approach using machine learning was proposed in the article. Initial tests confirmed the usefulness of neural networks with back-propagation of errors in predicting the value of feature *w*(11) on the basis of the features from *w*(1) to *w*(10). The proposed method was divided into two stages: training and testing. These two stages are directly related to the division of the entire database (the results obtained from 172 patients) into two sets: the training set containing 2/3 of the total number of patients, i.e. 113 patients, and the test set containing the remaining 1/3 of the patients, i.e. 59 patients. This type of division into the training and test set is typical of machine learning. In the present work, tests were conducted for neural networks with back-propagation of errors containing from 1 to 10 inputs and one output from the log-sigmoid transfer function. The networks provided one hidden layer. The tests were performed for different numbers of neurons in the hidden layer *s* from *s* = 10 to 500 every 10 neurons. The training process, unless stated otherwise, was performed in each case one million times. Every time after 1000 iterations, the order of individual patients in the training was random. In addition, every 1000 iterations, the error for the values obtained from the network $w_{i,j}^{s} \left( { 1 1} \right)$ was compared to ground truth—*w*_*i*_(11), defined as:

1 $$\delta \left( j \right) = \frac{1}{59}\mathop \sum \limits_{i = 1}^{59} \left| {w_{i,j}^{s} (11) - w_{ i} (11)} \right|$$

where: $w_{i,j}^{s} \left( { 1 1} \right)$—the value obtained from the neural network output for *i*-th patient and *j*-th randomisation, *w*_*i*_(11)—the value of ground truth for *i*-th patient, *s*—the number of neurons in the hidden layer.

**Fig. 2:**
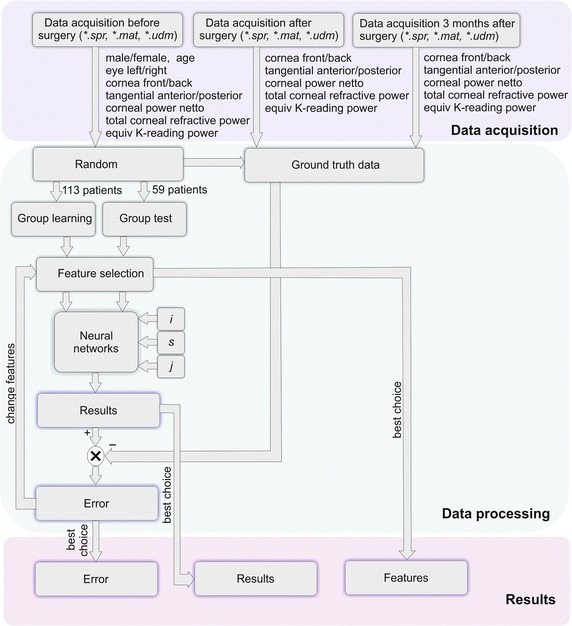
Block diagram of the proposed algorithm for data analysis. This diagram is divided into three areas: data acquisition including the data of patients before refractive surgery, 1 month after surgery and 3 months later; data analysis including training and modifications of the structure of neural networks; the results obtained when the best features were selected

Since there is no evidence in the available literature [43] to what extent the features from *w*(1) to *w*(10) affect the outcome—tests were conducted (a million iterations) for each possible combination of the features *w*. In total, several billion iterations were performed for different combinations of features, different randomisations of cases in the training and test vector, and for different numbers of neurons in the hidden layer. A block diagram of the entire processing is shown in Fig. 2.

**Fig. 3:**
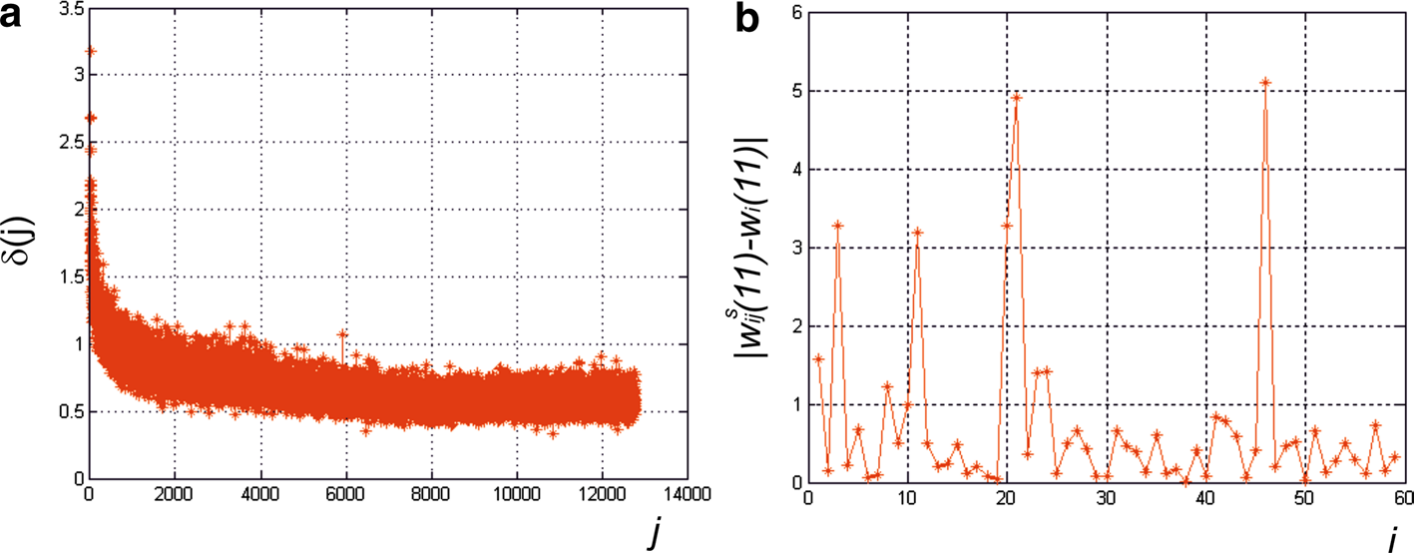
Sample results of network testing: **a** the values of the error *δ*(*j*) for *j* randomisations (*j*∈(1,12000) and *s* = 150) and the features in the training vector *w*(3), *w*(4) and *w*(5). Each randomisation and calculation of the error are performed after 1000 iterations of network training; **b** the absolute difference between the power of the cornea predicted by the neural network and the reference power (ground truth) for 59 test cases

Examples of the results of network testing are shown in Fig. 3. Figure 3a shows the values of the error *δ*(*j*) for *j* randomisations (*j*∈(1,12000) and *s* = 150) and the features in the training vector *w*(3), *w*(4) and *w*(5). In the case shown in Fig. 3a, each randomisation and calculation of the error is performed after 1000 iterations of network training. Examples of the differences between the power of the cornea predicted by the neural network and the compared power (ground truth—see Table 1) for 59 test cases are shown in Fig. 3b. The graphs in Fig. 3 confirm the need for additional measurement of the maximum value *δ*_*max*_ of the error *δ*(*j*) for *j* randomisations.

The minimum values are not significant from the point of view of analysis of results (since there is the mean value and standard deviation of the mean). This is due to the practical usefulness of the results of prediction and the attitude of patients who expect the same quality of vision (without glasses) as before cataract surgery.

Therefore, Table 3 shows the results of measurement of the mean value of the error *δ*(*j*) for *j* = *1000* randomisations, where for each randomisation there are 1000 iterations of network training and *s* = 150. The value of *s* equal to 150, which is the number of neurons in the hidden layer, was adopted on the basis of the measurements of the error *δ*(*j*) for all features. The relationships and participation of individual features were confirmed in the correlation matrix created for all features *w* which contributed to providing the best results (in accordance with Table 3).

**Table 3:** The first 10 smallest mean values of the error *δ* for 59 test patients for different configurations of features, *j* = 1000 randomisations and 1000 iterations for each randomisation and the number of neurons in the hidden layer *s* = 150

Feature (“0” absent, “1” present)										*δ* [D]	*δ*_*max*_ [D]
*w*(1)	*w*(2)	*w*(3)	*w*(4)	*w*(5)	*w*(6)	*w*(7)	*w*(8)	*w*(9)	*w*(10)		
0	0	0	0	1	1	1	0	0	1	0.16	0.75
0	0	0	0	0	1	1	0	0	1	0.19	0.72
0	1	0	1	1	1	1	1	1	0	0.19	0.8
0	0	0	1	0	1	0	1	0	1	0.29	0.8
0	0	0	0	0	0	1	1	0	1	0.36	0.9
0	1	0	0	1	1	0	0	0	1	0.36	1.1
0	0	0	1	1	0	0	1	1	1	0.37	0.8
0	0	0	1	1	0	1	1	1	1	0.39	0.8
0	0	0	0	1	0	1	1	1	1	0.40	1.1
0	1	0	1	1	0	1	1	0	1	0.40	1.2

As mentioned above, the tests were performed for different numbers of neurons in the hidden layer from *s* = 10 do 390 every 20 neurons. 500 iterations were performed every *j*-th randomisation. The graph in Fig. 5 shows changes in the error *δ*(*j*) as a function of different number of neurons in the hidden layer for all the features *w*. Detailed analysis of the results obtained and their interpretation are presented in the next section.

## Results and discussion

**Fig. 4:**
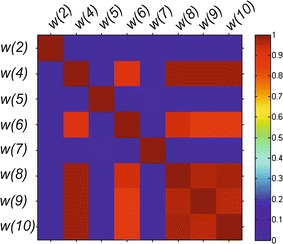
Correlation matrix created for all the features *w* participating in obtaining the best results in accordance with Table 3. The artificial *colour palette* has been added to facilitate reading of the correlation values

**Fig. 5:**
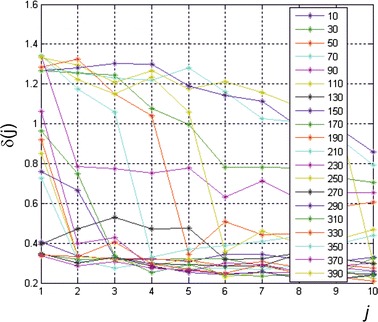
Graph of changes in the error *δ*(*j*) as a function of *different numbers* of neurons *s* in the hidden layer for all features *w*. The measurements were performed every 20 neurons for 500 iterations (*j* = 1)

The results obtained, especially Table 3 and Fig. 4, lead to some practical conclusions:

- the most common feature in the top 10 results is *w*(10), which is equiv K-reading power;
- the features *w*(1) and *w*(3) are not found in any of the 10 top results. In other words, the left/right eye and gender do not affect the obtained results and so they are not practically important for predicting the correct value of the optical power. A similar situation is with the patient’s age which is taken into consideration only in three cases (see Table 3);
- the smallest number of features involved in correct corneal power calculation (with an error *δ* of less than 0.4 dioptres) is 3. These are the following combinations of features: *w*(6), *w*(7) and *w*(10) as well as *w*(7), *w*(8) and *w*(10). Medical interpretation of occurrence of these features results directly from the participation of tangential anterial/posterior, net power of the cornea, and equiv K-reading power in correct corneal power calculation [the feature *w*(11)];
- the number of neurons in the hidden layer ranging from 10 to 590 provides the same accuracy of cornea power prediction (Fig. 5). There is only a change in the time of network training—the value of the variable *j* (for the same number of iterations).

**Fig. 6:**
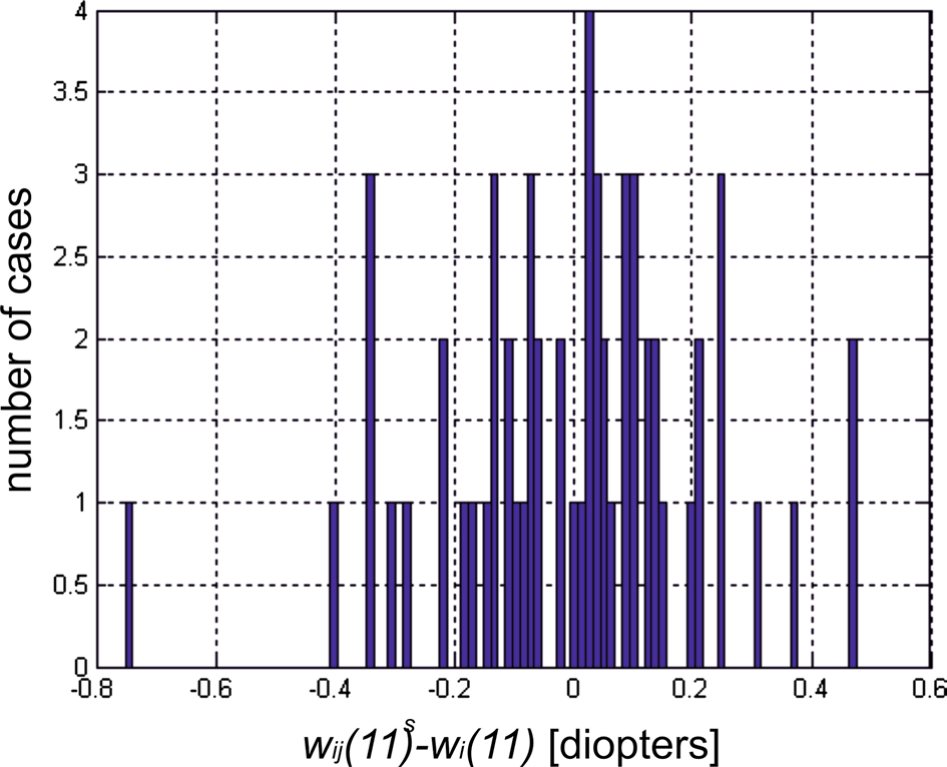
*Histogram* obtained for 59 test patients for network configurations providing the best results. The values on the *x-axis* are the differences between the prediction results obtained using neural networks with back-propagation of errors and the ground truth values, whereas the values on the *y-axis* refer to the number of cases (number of patients)
- the residual difference in corneal power evaluation obtained lead to no clinically significant variation of IOL to implant in eye facing cataract surgery after myopic refractive surgery.
- analysis time for a single patient (single eye) for PC with Intel^®^ Xeon^®^ X5680 3.33 GHz CPU in Matlab^®^ Version 7.11.0.584 (R2010b) with Signal Processing Toolbox Version 7.1 (R2010b), Neural Network Toolbox 7.0 (R2010b) and Statistics Toolbox (R2010b) does not exceed 0.1 s. At the same time, the time of network training is about 3 s for 1000 iterations (when the number of neurons in the hidden layer *s* = 400).

The results can be compared to the results obtained by other authors. The publications on estimating the exact corneal power offer various formulas and corrections to be afterwards applied to the lens power calculation. Previous published papers about same topic showed corneal power evaluation after refractive surgery not to be reliable, presenting a wide range of errors, depending by the device tested and the study population [1–4, 9, 10, 27]. Compared to the results obtained in this study:

- the presented methods are significantly different from each other in terms of mathematical formulas, which confirms that the artificial intelligence approach presented in this paper is correct;
- the error obtained when using neural networks stems from the measurement noise which in turn results from the applied measurement method. Therefore, there is no possibility of analytical (assuming a random nature of methodology errors) reduction of the residual error in corneal power prediction to a value below 0.16 ± 0.14 dioptres. It is also impossible by using artificial intelligence—a histogram in Fig. 6 shows the exact value of the difference between prediction results obtained from neural networks with back-propagation of errors and the ground truth values;

On this basis, it is possible to formulate the following conclusions presented in the next section.

## Conclusions

The new approach to predicting the corneal power in patients previously undergone myopic refractive surgery can be characterized in the following points:

- the proposed method of corneal power prediction is fully automatic and does not require operator intervention;
- the presented method provides for 172 test patients (113 patients forming the training set and 59 patients in the test set) the error of corneal power prediction at the level of 0.16 ± 0.14 dioptres with a maximum value of 0.75 dioptres;
- the presented method provides the above results for the following three input parameters (obtained with the Pentacam): tangential anterial/posterior, corneal net power and equiv K-reading power.

Corneal power prediction for IOL calculation in patients who had previously undergone surgical correction of myopic refractive defects should be made the same way as in relation to the optical measurement path. On the one hand, the applied approach and mathematical relationships implemented in the Penatacam tomograph require correction. Such correction is proposed by many authors. On the other hand, as shown in this article, artificial intelligence in the form of neural networks enables to reduce significantly the prediction error. Regardless of the results obtained, there is also the aforementioned random noise resulting from resolution and non-linearity errors (and also the measurement method itself). Thus, the presented method of artificial intelligence does not exhaust this very interesting topic which has many practical applications.

## Abbreviations

ILM: internal limitans membrane
RPE: retinal pigment epithelium
D: dioptre
std: standard deviation of the mean
